# Influence of functional dentition on satisfaction with oral health and impacts on daily performance among Brazilian adults: a population-based cross-sectional study

**DOI:** 10.1186/s12903-017-0402-5

**Published:** 2017-07-11

**Authors:** Loliza Luiz Figueiredo Houri Chalub, Raquel Conceição Ferreira, Andréa Maria Duarte Vargas

**Affiliations:** Department of Community and Preventive Dentistry, School of Dentistry, Federal University of Minas Gerais, Antônio Carlos Avenue, 6627 – Campus – CEP, Belo Horizonte, Minas Gerais 31270-911 Brazil

**Keywords:** Functional dentition, Satisfaction, Oral impact daily performance, Tooth loss, Oral health-related quality of life

## Abstract

**Background:**

Dental esthetics, chewing and speech should be preserved in a dentition denominated *functional* and are closely related to satisfaction with oral health (SOH), impacts caused by oral problems and have a possible association with Oral Health-Related Quality of Life. Thus, the purpose of the present study was to investigate the influence of different concepts of functional dentition (FD) on both SOH and impacts on daily performance (IDP) among Brazilian adults.

**Methods:**

A cross-sectional study was conducted with 9564 adults (35–44 years). SOH and IDP were evaluated using the Oral Impacts on Daily Performance (OIDP) questionnaire. FD was considered based on four different definitions: I-classification of the World Health Organization (FDWHO = ≥20 teeth); II-well-distributed teeth (WDT = ≥10 teeth in each arch); III-classified by esthetics and occlusion (FD_Class5_ = sequential presence of one tooth in each arch, ≥10 teeth in each arch, 12 anterior teeth, ≥three posterior occluding pairs [POPs] of premolars and ≥one POP molar bilaterally); and IV-classified by esthetics, occlusion and periodontal status (FD_Class6_ = FD_Class5_ plus all sextants with CPI ≤ 3 and/or CAL ≤ 1). The proportion of adults satisfied with oral health and without overall impact (OIDP = 0) was calculated for each definition of FD. Multiple Poisson regression models were adjusted by demographic-socioeconomic characteristics, self-reported oral problems and the use of dental services for each dependent variable.

**Results:**

When FD_Class5_ and FD_Class6_ were considered a greater proportion of adults reported being satisfied (52.1 and 53.1%, respectively) and have OIDP = 0 (52.4 and 53.3, respectively). In the multiple models, SOH was associated with FD_Class5_ (RP = 1.21) and FD_Class6_ (RP = 1.24) and OIDP = 0 was associated with WDT (RP = 1.14) and FD_Class6_ (RP = 1.21).

**Conclusions:**

The greater influence of WDT, FD_Class5_ and FD_Class6_ on aspects related to quality of life in comparison to FDWHO demonstrates the need for the establishment of a broader definition of FD that encompasses subjective aspects.

## Background

The self-perception of dental status and oral function is an important aspect of oral health. Patient satisfaction should be one of the main goals when planning oral health care [1]. The evaluation of health status and treatment results should involve the impact of an adverse condition, its treatment and its consequences with regard to quality of life. Clinical indicators alone are no longer recognized as sufficient for describing the health status of individuals or populations [2]. Thus, the assessment of oral health-related quality of life (OHRQoL) has become the object of studies in the field of dentistry [1–6].

The Oral Impacts on Daily Performance (OIDP) questionnaire is one of the most widely used assessment tools for OHRQoL. This measure is founded on a theoretical protocol derived from a modification of the International Classification of Impairments, Disabilities and Handicaps of the World Health Organization (WHO), for which the adaptation to dentistry was performed by Locker [7]. The OIDP evaluates oral impacts on behavior and the ability to perform key day-to-day activities, which are grouped into dimensions (physical, psychological and social performance). The OIDP test and validation study found that 73.6% of individuals had at least one daily activity affected by oral impact in the previous 6 months [8], the most affected of which were eating, emotional stability and smiling. Using the OIPD, several studies have identified eating as one of the activities most affected by oral problems [9–11].

Clinical conditions, such as the number of functional, decayed or missing teeth [8, 11], chewing ability [5], tooth mobility, periodontal attachment loss and missing anterior and posterior teeth [10], exert impacts on daily performance. Moreover, socioeconomic status, the use of dental services and demographic characteristics also affect the OIDP score [5, 10, 11]. A reduced dentition, such as a shortened dental arch (SDA) and having a functional dentition (FD), is also potentially associated with OHRQoL, which has been explored little in the literature [1, 3, 12, 13]. Dental esthetics, chewing and speech, which should be preserved in a dentition denominated *functional*, are closely related to satisfaction with oral health (SOH) and impacts caused by oral problems and have a possible association with OHRQoL. The small number of investigations into this issue may be due to the lack of a consensus on the definition of FD.

For the WHO, FD is the retention of a natural, esthetic, functional dentition of no less than 20 teeth throughout life with no need for tooth replacement [14]. However, the number of teeth alone seems to be too simplistic to describe oral health status in terms of functionality. There is some evidence that teeth also need to be well distributed (at least 10 teeth in each arch) to ensure adequate oral function [15]. But the concept of well-distributed teeth (WDT) remains primarily quantitative because it does not take into account that each tooth group performs a different oral function. Nguyen et al. [16] developed a functional classification system for dentitions based on five sequential, hierarchical levels, which involve the following requirements: 1) at least one tooth in each arch; 2) at least ten teeth in each arch; 3) all anterior teeth; 4) three or four posterior occluding pairs (POPs) of pre-molars; and 5) at least one molar POP bilaterally. This novel dental configuration for defining FD has been employed in studies involving populations in countries in Europe and Southeast Asia [1, 3, 16]. The system was first employed in the Americas by Chalub et al. [17, 18] and the dentition that takes into account all levels of the system originated a new definition of FD denominated FD_Class5_ by these authors [17].

When the criteria of this classification system are present in dentitions, positive impacts are found with regard to chewing both fibrous and pasty foods [19, 20], greater satisfaction with one’s mouth [1] and better OHRQoL [3]. However, this system does not include periodontal status in the definition of FD. The incorporation of this aspect is justified by knowledge that the loss of periodontal support tissue exerts a negative effect on chewing ability [21], which is one of the most important oral functions. Moreover, signs and symptoms of periodontal disease, such as periodontal pockets ≥5 mm, swelling, pain and halitosis, have demonstrated an association with poorer OHRQoL in patients undergoing periodontal treatment [22, 23]. Better oral health and regular follow up of patients submitted to periodontal support therapy are also reflected in fewer impacts on daily performance (IDP) measured using the OIDP [24]. By including the periodontal criterion to the system proposed by Nguyen et al. [16], a definition of FD that encompasses criteria addressing esthetics, occlusion and periodontal status was established, denominated FD_Class6_ by the authors [17].

The present study was conducted based on the belief that periodontal status plays an important role in the establishment of FD and due to the literature shortage about the relationship between different definitions of the FD and both SOH and IPD among Brazilian adults. To the best of our knowledge there is only one population-based study comparing OHRQoL between Brazilian adults having SDA and those with more teeth [13]. Despite this, the study cited [13] did not compare the relationship of so many FD definitions with OHRQoL like ours study did. The findings are expected to contribute to the establishment of a new definition of FD that encompasses clinical normative and subjective aspects. Thus, the aim of the present study was to investigate the influence of different concepts of functional dentition (FD) on both SOH and impacts on daily performance (IDP) among Brazilian adults.

## Methods

### Study design and sampling procedures

The data employed in this study were obtained from the 2010 National Oral Health Survey (NOHS) conducted by the Brazilian Health Ministry [25]. The division of the country into five large regions (north, northeast, southeast, south and central west) was determined by the Brazilian Institute of Geography and Statistics and has been adopted in epidemiological studies with a national scope. Thus, the sampling plan involved these regions as domains, along with the capitals of the 27 states, including the Federal District, which totaled 32 domains formed by 177 municipalities (27 capitals and 30 municipalities in each region). The sample was determined with the random selection of municipalities and census sectors, configuring multi-stage cluster sampling with probability proportional to size [26]. Detailed information on the method is found elsewhere [26, 27].

For the 35-to-44-year-old age group used in the present study, the calculation of the sample size was based on the mean of the number of decayed, missing and filled teeth (DMFT) index in each domain using data from the national survey conducted in 2003 [28]. The values were multiplied by 2 to account for the design effect and corrected to compensate for a possible 20% loss rate [26].

### Data acquisition

Oral examinations were performed based on the WHO guidelines for epidemiological studies [29] using the DMFT index as well as the community periodontal index (CPI) and clinical attachment loss (CAL) for the study of dental caries and periodontal status, respectively. Among the clinical data, only the number of teeth present (including 3rd molars) and periodontal data (CPI and CAL) for sextants were considered in the analyses. The total number of teeth was determined by the sum of the number of teeth present, excluding codes 4, 5 (missing) and 8 (non-erupted) of the DMFT index. A POP was defined as a pair of antagonist posterior teeth on each side of the mouth, for example, the pairs formed by teeth 16 and 46 and teeth 26 and 36. Periodontal status was determined by the highest CPI and CAL codes found among the sextants. Advanced periodontal disease was defined as the presence of deep pockets (CPI = 4) or excluded sextant and CAL equal to or greater than 6 mm (CAL ≥ 2) or excluded sextant in at least one sextant of the mouth, based on diagnostic criteria described in the literature [30].

Interviews were held on SOH, IDP, demographic-socioeconomic characteristics, self-reported oral problems and the use of dental services. The field teams were trained and calibrated for each age group and problem studied (acceptable minimum limit for weighted Kappa: 0.65) [27].

### Dependent variables

SOH was determined using the following question: “With regard to your teeth/mouth, are you very satisfied, satisfied, neither satisfied nor dissatisfied, dissatisfied or very dissatisfied (or doesn’t know/didn’t answer)?” [31]. The responses were dichotomized as unsatisfied (neither satisfied nor dissatisfied, dissatisfied and very dissatisfied categories) or satisfied (satisfied and very satisfied categories). “Neither satisfied nor dissatisfied” was included in the unsatisfied classification due to the belief that indifference reported by adults approaches a lack of satisfaction more than satisfaction, especially with regard to oral factors. The variables used to measure IDP were determined based on the assertion and questions shown in Table 1.

**Table 1:** Issues related to nine performances that compose the Oral Impacts on Daily Performances questionnaire

Some people have problems that are caused by their teeth. Among the situations listed below, which apply to your experiences in the last 6 months?
1) Had difficulty eating because of your teeth or felt tooth pain (dental sensitivity) when drinking cold or hot liquids (no, yes or doesn’t know/didn’t answer)
2) Your teeth causes you discomfort when brushing (no, yes or doesn’t know/didn’t answer)
3) Your teeth made up upset or irritable (no, yes or doesn’t know/didn’t answer)
4) Did not go out, have fun, go to parties or go on trips because of your teeth (no, yes or doesn’t know/didn’t answer)
5) Did not practice sports because of your teeth (no, yes or doesn’t know/didn’t answer)
6) Had difficulty speaking because of your teeth (no, yes or doesn’t know/didn’t answer)
7) Your teeth made you embarrassed to smile or speak (no, yes or doesn’t know/didn’t answer)
8) Your teeth had a negative effect on studying/doing housework/working (no, yes or doesn’t know/didn’t answer)
9) Did not sleep or slept poorly because of your teeth (no, yes or doesn’t know/didn’t answer)

The items refer to nine performances evaluated using a modified version of the OIDP index where each item had response options of “no” (scored as 0), “yes” (scored as 1) or “doesn’t know/didn’t answer”. The final OIDP score resulted from the sum of the scores for each performance. Subsequently, another dichotomous variable was generated: absence of impact (total OIDP = 0) or presence of impact (total OIDP ≥ 1). Performances with impact frequencies related to the teeth higher than 20% constituted separate dependent variables. Internal consistency of OIDP assessed through Cronbach’s alpha coefficient was 0.816 and ranged from 0.761 ([11 years of schooling) to 0.830 (5–8 years of schooling), suggesting that OIDP reliability was not influenced by the different levels of education of participants [11].

### Main independent variables

The main independent variables were four definitions of FD:

- FDWHO: the presence of 20 or more teeth – established by the WHO in the scope of global goals in oral health [14];
- WDT: based on the concept of 20 “well-distributed teeth”, which establishes at least 10 in each arch [15, 16];
- FD_Class5_: classified by esthetics and occlusion – sequential presence of one tooth in each arch ➔ ≥ 10 teeth in each arch ➔ 12 anterior teeth ➔ three premolar POPs ➔ ≥ one molar POP bilaterally [16];
- FD_Class6_: classified by esthetics, occlusion and periodontal status, corresponding to the same conditions as FD_Class5_ plus all sextants with CPI ≤ 3 and/or CAL ≤ 1 [17].

The latter two definitions result from the functional classification system of dentitions adapted from Nguyen et al. [16]. A complete description and evaluation of this system for Brazilian adults can be found in previous publications [17, 18].

### Controlling independent variables

The controlling variables were socioeconomic status (monthly household income and schooling), self-reported oral problems (self-rated need for treatment/dentures and toothache/dental pain in previous 6 months), use of dental services (dental appointment at least once in life, type of service utilized for last dental appointment and reason for last dental appointment) and demographic characteristics (gender and self-declared skin color).

### Statistical analysis

Descriptive analysis of the variables was performed for the characterization of the sample. Estimates of the prevalence of SOH and IDP and respective confidence intervals (95% CIs) were calculated for the entire sample and for each category of the independent variables. The calculations were weighted by the sampling weight to account for the design effect (complex sampling) using the Complex Samples command of the SPSS program. The percentages of individuals satisfied with their oral health, without overall impact (OIDP = 0) and without impact on eating, brushing teeth, emotional state or smiling/speaking were calculated in relation to the entire sample. These calculations were generated following dichotomization (presence/absence of the criterion) based on the cutoff point for each level of the FD classification system adapted from Nguyen et al. [16], considering the sequential nature of the levels [17]. The results were represented in bar graphs and the significance of the differences between percentages after dichotomization was determined based on respective 95% CIs. Multiple Poisson regression models were created for each dependent variable. The definitions of FD were incorporated separately in the multiple models with controlling variables. Associations were considered significant at a 5% probability level (*p* ≤ 0.05). All statistical analyses were performed with the aid of the SPSS® 17.0 program (SPSS Inc., Chicago, IL, USA) and graphs were created using the Microsoft Excel® 2013 program.

## Results

A total of 9564 individuals composed the final sample (examinations not performed on 215). The majority of adults reported not being satisfied with their teeth and mouth (58.9%; 95% CI: 55.7–62.0) and had impact on at least one daily activity (OIDP ≥ 1) (55.5%; CI 95%: 51.2–59.7). Difficulty eating/dental sensitivity, discomfort when brushing, influence on emotional state, embarrassed to smile/speak had impact frequencies greater than 20%. The prevalence of FD varied with the definition: 77.9% for FDWHO, 72.9% for WDT, 42.6% for FD_Class5_ and 40.3% for FD_Class6_ (Table 2).

**Table 2:** Distribution of Brazilian adults in accordance to categories of dependent and independent variables, 2010

Variables (*n*)	Percent	95% CI
*Dependent variables*		
Satisfaction with oral health (9505)		
Unsatisfied	58.9	55.7–62.0
Satisfied	41.1	38.0–44.3
Total OIDP (9550)		
OIDP = 0	44.5	40.3–48.8
OIDP ≥1	55.5	51.2–59.7
Difficulty eating or dental sensitivity (9524)		
Yes	33.5	30.3–36.9
No	66.5	63.1–69.7
Discomfort when brushing (9531)		
Yes	26.6	23.4–29.9
No	73.4	70.1–76.6
Influence on emotional state (9521)		
Yes	25.8	22.8–29.2
No	74.2	70.8–77.2
Influence on going out (9533)		
Yes	15.4	13.4–17.6
No	84.6	82.4–86.6
Influence on practicing sports (9507)		
Yes	6.3	4.7–8.3
No	93.7	91.7–95.3
Difficulty speaking (9540)		
Yes	14.5	12.6–16.7
No	85.5	83.3–87.4
Embarrassed to smile/speak (9530)		
Yes	27.3	24.4–30.3
No	72.7	69.7–75.6
Difficulty studying, working, doing chores (9525)		
Yes	11.4	9.6–13.4
No	88.6	86.6–90.4
Difficulty sleeping (9516)		
Yes	18.7	16.2–21.5
No	81.3	78.5–83.8
*Independent variables*		
FDWHO (9564)		
Present	77.9	75.4–80.2
WDT (9564)		
Present	72.9	70.1–75.4
FD_Class5_ (9564)		
Present	42.6	40.0–45.2
FD_Class6_ (9392)		
Present	40.3	37.7–43.0
Monthly household income (9337)		
≤ US$284	13.0	11.1–15.0
US$285 - US$852	53.4	49.4–57.3
US$853 - US$2557	29.9	26.8–33.1
> US$2557	3.8	2.5–5.8
Schooling (9495)		
Up to 4 years	20.7	17.9–24.0
5 to 8 years	28.7	26.4–31.2
9 to 11 years	28.8	26.3–31.5
*Independent variables*		
Self-rated need for treatment (9359)		
Yes	77.7	75.3–79.9
No	22.3	20.1–24.7
Toothache/dental pain (9495)		
Yes	27.9	25.4–30.6
No	72.1	69.4–74.6
Self-rated need for dentures or to change dentures (9287)		
Yes	36.0	32.8–39.2
No	64.0	60.8–67.2
Dental appointment at least once in life (9509)		
Yes	6.9	5.2–9.0
No	93.1	91.0–94.8
Type of service utilized for last dental appointment (8812)		
Public	37.7	33.5–42.1
Private	49.8	46.0–53.7
Health insurance, partnerships, others	12.5	11.0–14.2
Reason for last dental appointment (8803)		
Extraction, pain	31.1	28.7–33.7
Treatment	44.7	41.5–48.0
Checkup, prevention, others	24.2	21.6–26.9
Gender (9564)		
Female	63.4	60.1–66.6
Male	36.6	33.4–39.9
Self-declared skin color (9564)		
Black	10.8	9.3–12.6
White, yellow, brown, indigenous	89.2	87.4–90.7

*n* number – sample size, *%* percentage, *CI* confidence interval, *OIDP* Oral Impacts on Daily Performance, *FDWHO* ≥ 20 teeth present, *WDT* ≥ 10 teeth in each arch, *FD*_*Class5*_ functional dentition classified by occlusion and esthetics, *FD*_*Class6*_ functional dentition classified by occlusion, esthetics and periodontal status

FD was generally associated with both SOH and IDP, independently of the definition employed. Only FDWHO was not associated with SOH (Table 3). Presence of functional dentition (based on the four definitions) were associated with absence of impacts on eating/dental sensitivity, on going out, on speaking and on being embarrassed to smile/speak. Presence of FD_Class5_ and FD_Class6_ also were associated with absence of impacts on emotional state and on studying, working, doing chores (Table 4).

**Table 3:** Satisfaction with oral health and impacts on daily performance in accordance to categories of independent variables among Brazilian adults, 2010

Variables		Dependent variables			
		Satisfaction with oral health - satisfied		Total OIDP = 0	
Independent variables		%	95% CI	%	95% CI
FDWHO	Absent	34.9	30.1–40.0	**36.7**	32.6–41.0
	Present	42.9	39.3–46.5	**46.7**	41.8–51.6
WDT	Absent	**34.2**	29.4–39.5	**35.6**	31.3–40.1
	Present	**43.7**	39.8–47.5	**47.8**	42.9–52.8
FD_Class5_	Absent	**33.0**	29.4–36.7	**38.6**	34.5–43.0
	Present	**52.1**	47.9–56.2	**52.4**	47.1–57.7
FD_Class6_	Absent	**32.7**	29.3–36.4	**38.3**	34.1–42.7
	Present	**53.1**	48.9–57.3	**53.3**	48.1–58.5

*OIDP* Oral Impacts on Daily Performance; *%* percentage; *CI* confidence interval; *FDWHO* ≥ 20 teeth present; *WDT* ≥ 10 teeth in each arch; *FD*_*Class5*_ functional dentition classified by occlusion and esthetics; *FD*_*Class6*_ functional dentition classified by occlusion, esthetics and periodontal status; *data with non-overlapping 95% CI in bold

**Table 4:** Absence of impacts measured by the Oral Impacts on Daily Performance according to categories of independent variables among Brazilian adults, 2010

Independent variables		Dependent variables																	
		Difficulty eating or dental sensitivity (no)		Discomfort when brushing (no)		Influence on emotional state (no)		Influence on going out (no)		Influence on practicing sports (no)		Difficulty speaking (no)		Embarrassed to smile/speak (no)		Difficulty studying, working, doing chores (no)		Difficulty sleeping (no)	
		%	95% CI	%	95% CI	%	95% CI	%	95% CI	%	95% CI	%	95% CI	%	95% CI	%	95% CI	%	95% CI
FDWHO	Absent	**59.8**	55.5–63.9	72.2	67.5–76.4	69.0	64.1–73.6	**79.8**	75.9–83.2	93.0	90.6–94.8	**75.4**	71.3–79.1	**61.6**	56.9–66.2	85.3	81.6–88.3	81.3	77.6–84.5
	Present	**68.4**	64.4–72.1	73.8	70.0–77.3	75.6	72.2–78.7	**85.9**	83.4–88.1	93.9	91.4–95.7	**88.3**	86.1–90.3	**75.9**	72.4–79.0	89.6	87.1–91.6	81.3	78.3–84.0
WDT	Absent	**58.0**	53.1–62.8	71.4	66.5–75.9	**68.2**	63.1–72.9	**79.7**	76.1–82.8	92.0	89.7–93.8	**75.9**	72.1–79.4	**60.1**	55.2–64.9	84.8	80.8–88.1	80.4	76.5–83.9
	Present	**69.6**	65.8–73.2	74.2	70.4–77.7	**76.4**	73.0–79.4	**86.4**	83.8–88.7	94.3	91.6–96.2	**89.0**	86.8–90.9	**77.4**	74.0–80.5	90.0	87.5–92.1	81.6	78.6–84.3
FD_Class5_	Absent	**61.5**	57.8–65.1	71.7	67.8–75.3	**70.2**	66.4–73.7	**81.4**	78.6–83.9	92.5	90.6–94.0	**80.1**	77.3–82.7	**64.3**	60.5–68.0	**85.4**	82.5–87.9	79.3	76.1–82.1
	Present	**73.1**	68.7–77.2	75.8	71.6–79.5	**79.5**	75.4–83.0	**88.8**	85.4–91.5	95.4	91.9–97.4	**92.7**	90.2–94.6	**84.0**	81.0–86.6	**93.0**	90.7–94.7	84.0	80.7–86.9
FD_Class6_	Absent	**61.3**	57.6–64.9	71.2	67.2–75.0	**69.9**	65.8–73.7	**80.7**	77.9–83.3	**91.9**	89.7–93.8	**80.1**	77.3–82.6	**63.9**	60.2–67.5	**84.7**	81.3–87.5	79.0	76.0–81.7
	Present	**73.9**	69.6–77.7	76.5	72.2–80.4	**80.2**	76.4–83.5	**90.1**	87.4–92.2	**96.1**	93.9–97.6	**93.2**	90.8–95.0	**85.2**	82.1–87.9	**94.1**	92.2–95.6	84.5	81.2–87.3

*%* percentage, *CI* confidence interval, *FDWHO* ≥ 20 teeth present, *WDT* ≥ 10 teeth in each arch, *FD*_*Class5*_ functional dentition classified by occlusion and esthetics, *FD*_*Class6*_ functional dentition classified by occlusion, esthetics and periodontal status; *data with non-overlapping 95% CI in bold

**Fig. 1:**
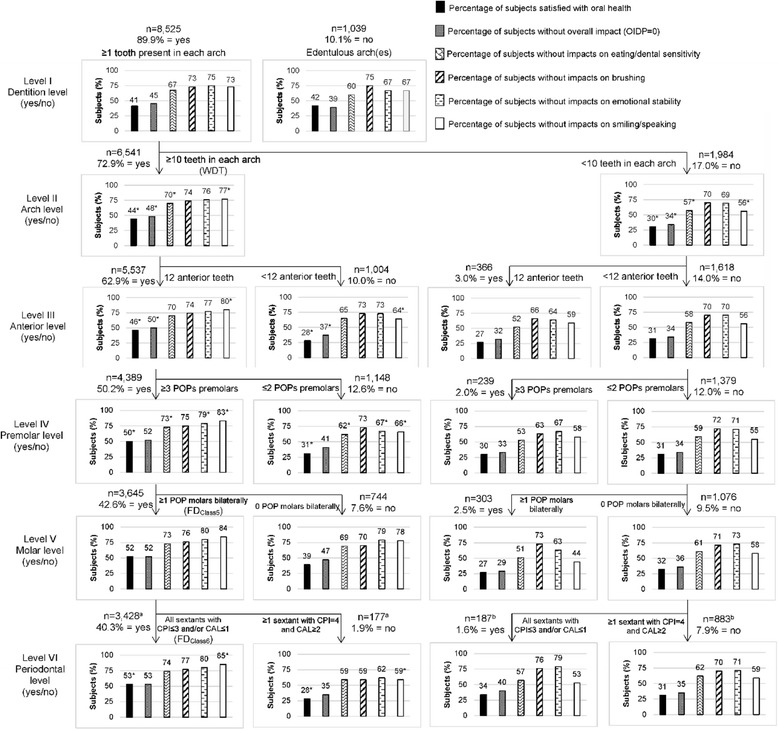
Satisfaction with oral health, overall impact and impacts on daily performances in accordance to the functional dentition classification system. *Asterisk indicates significant differences in the percentage of subjects within the same level of the system of each branch (“≥10 teeth in each arch” and “<10 teeth in each arch”)

The percentage of individuals who were satisfied with their oral health, those without impact, those without impact on eating and those without impact on smiling/speaking was significantly higher among adults with WDT (44%, 48%, 70% and 77%, respectively) than those without WDT (30%, 34%, 57% and 56%, respectively). A significant influence of the criteria of the six levels (except level V) was found on SOH and IDP only on the left branch of the figure (WDT present). The percentage of adults satisfied with their oral health (53%) and not embarrassed to smile/speak (85%) among those with FD_Class6_ was larger than the percentage of those with FD_Class5_ (28 and 59%, respectively) (Fig. 1).

Table 5 displays the results of the multiple Poisson regression models for SOH and IDP (overall impact and impacts on eating, brushing, emotional state and smiling/speaking). FD_Class5_ and FD_Class6_ were the definitions associated with higher prevalence rates of satisfied adults (21 and 24%, respectively). WDT and FD_Class6_ were associated with an absence of overall impact (OIDP = 0). All FD definitions were positively and significantly associated with smiling/speaking.

**Table 5:** Prevalence ratios of definitions of functional dentition in multiple Poisson regression models for satisfaction with oral health, overall impact and impacts on eating, brushing, emotional state and smiling/speaking among Brazilian adults, 2010

Variables		Dependent variables^a^											
		Satisfaction with oral health - satisfied		Total OIDP = 0		Difficulty eating or dental sensitivity (no)		Discomfort when brushing (no)		Influence on emotional state (no)		Embarrassed to smile/speak (no)	
Independent variables		PR	*p*	PR	*p*	PR	*p*	PR	*p*	PR	*p*	PR	*p*
FDWHO	Present	1.05	0.573	1.10	0.136	1.01	0.880	0.98	0.696	1.06	0.131	**1.14**	0.005
WDT	Present	1.13	0.238	**1.14**	0.037	1.07	0.427	0.99	0.773	1.07	0.056	**1.20**	<0.001
FD_Class5_	Present	**1.21**	0.023	1.10	0.077	1.03	0.451	0.94	0.073	1.04	0.471	**1.19**	<0.001
FD_Class6_	Present	**1.24**	0.009	**1.11**	0.026	1.02	0.600	0.96	0.190	1.00	0.963	**1.20**	<0.001

*OIDP* Oral Impacts on Daily Performance; *PR* Prevalence Rate; *CI* confidence interval; *FDWHO* ≥ 20 teeth present; *WDT* ≥ 10 teeth in each arch; *FD*_*Class5*_ functional dentition classified by occlusion and esthetics; *FD*_*Class6*_ functional dentition classified by occlusion, esthetics and periodontal status ^a^ Individual multiple models for each dependent variable including each dental condition separately and controlling variables (monthly household income, schooling, self-rated need for treatment, toothache/dental pain in previous 6 months, self-rated need for dentures or to change dentures, reason for last dental appointment and gender); *significant values in bold (*p* ≤ 0.05)

## Discussion

Four definitions of FD were associated with SOH and IDP among Brazilian adults. However, the multiple models controlled for potential confounding variables revealed that only the broader definitions (WDT, FD_Class5_ and FD_Class6_) remained associated with SOH and an absence of overall impact. These finding support the defense of a broader definition of FD over that recommended by the WHO, which is based only on a quantitative criterion [16, 17]. This is the only population-based study involving adults conducted to evaluate the influence of four definitions of FD on both SOH and IDP.

Reduced dentitions used to define FD constitute the minimum number of teeth [2, 32] or SDA [12, 13, 33]. A few investigations have employed the functional classification system of dentitions proposed by Nguyen et al. [16] and have evaluated its effect on OHRQoL [1, 3], but periodontal status is not considered in such studies. Since the differences in OHRQoL between adults with SDA and those with more teeth could be attributable to variations in dental conditions such as periodontal disease [13] the inclusion of this criterion by our study endorses its importance.

Studies that have evaluated the influence of periodontal disease and treatment on quality of life have contributed considerably to current knowledge, but were not performed involving samples with population representativeness [22–24]. Nonetheless, the present investigation has limitations due to the use of secondary data. The clinical determination of occlusal contact between pairs of antagonist teeth (POPs) was not possible. Moreover, periodontal status was only recorded for tooth indices, as recommended by the WHO for epidemiological studies [29]. The normative evaluation on need to use or exchange prostheses was performed during the 2010 NOHS, but the criteria of the examination did not enable the identification of which teeth or regions of teeth were replaced by dentures, like it was done by Zhang et al. [3]. Thus, it was not possible to report the number/effect of dentures used by the subjects on their satisfaction and perceived oral impact. Another limitation of the NOHS is that no investigation of pain related to temporomandibular disorders (TMD) was performed. Missing posterior support could be associated with TMD pain. However, this limitation is relative, since a random clinical trial reports that the presence of a SDA is not a major risk factor for TMD pain when compared to molar replacement with removable dental prostheses [34]. Furthermore, caries was not included in the new definitions of functional dentition (FD_Class5_ and FD_Class6_), but the models were adjusted for the presence of toothache, which is one of the main consequences of caries. Therefore, this condition was considered by the presence of the symptom.

As in a previous study [1], the OIDP was employed using a non-validated method (dichotomous response option [no/yes]), which may constitute a limitation. However, internal consistency of OIDP assessed through Cronbach’s alpha coefficient was satisfactory (0.816) [11]. The validity of the dichotomous’ response approach is implicit in the significant associations between IDP and the FD definitions, treatment needs and toothache/dental pain. This inference is in accordance with the study that validated the OIDP for use on Brazilian adults, in which inverse correlations were found between the OIPD score and the perception of treatment need and SOH when the criterion validity and construct validity of the questionnaire were investigated [6]. However, another limitation is that the content and construct validation and reliability testing of the SOH question was not performed by the NHOS. Besides that, as the authors’ greater interest was to evaluate the effect of different definitions of functional dentition on the satisfaction of the participants with their oral health, the neutral category “neither satisfied nor dissatisfied” was added to the categories “dissatisfied” and “very dissatisfied” which also could be seen as a limitation.

The percentage of adults unsatisfied with their oral health in the present study (≈59%) was higher than that found for adults in Bulgaria (52%) with regard to general aspects, esthetics and chewing function [1]. However, the study cited differs from the present investigation due to the inclusion of a broader age range (≥20 years), a sample composed predominantly of males and individuals with medium-high schooling and the fact that all percentages were calculated only in relation to the total number of dentate individuals. Concerning the predominance of the female gender in the present study, this was expected, as women seem prone to more demanding self-evaluations, especially in relation to health conditions. Females were more likely to have a higher mean of OIDP extent compared to males and female gender predicted eating, cleaning, smiling, emotional status and social contact. Both results were adjusted for socio-economic variables and clinical oral health measures [11].

The prevalence of OIDP ≥ 1 was similar to rates reported for other populations [6, 9, 10], but lower than that in the study conducted to test and validate the questionnaire [8] and much higher than that found among Norwegian adults [4]. The context of these populations may partially be an explanation for this. The Brazilian adults in the present study likely shared cultural, socioeconomic and clinical characteristics more similar to those in southern Brazil [6], Tanzania [9] and Thailand [10] than adults in Norway [4], where living conditions are better. However, this is not the only explanation, since Adulyanon et al. [8] also conducted a study in Thailand, but with younger individuals than those evaluated by Srisilapanan and Sheiham [10] and report lower frequencies of dental caries and the use of dental services [8]. All this may have contributed to a more demanding perception of oral on daily performances [8].

Eating was the most frequently impacted daily performance, which is in agreement with data described in previous studies [4–6, 8–10] and was predictable, as chewing ability is associated with OHRQoL and wellbeing [35]. A dentition with many missing teeth can limit food consumption and nutrient intake, thereby affecting nutritional status [36]. Despite the greater impact on eating in comparison to the other aspects of the OIDP (≈34%), the frequency was lower than that reported in previous studies [5, 8–10], possibly due to a confounding factor, as the same question that addressed difficulty eating also addressed dental sensitivity [25].

Smiling/speaking was the second most affected item (≈27%), which is in agreement with findings of another studies [6, 8]. Smiling and speaking are important oral functions that play a role in social inclusion. Moreover, esthetic aspects of the teeth are increasingly valued, especially among Brazilians. This importance becomes clear when analyzing Fig. 1, which demonstrates that adults with WDT and a complete anterior region had a significantly higher SOH and absence of overall impact (OIDP = 0) in comparison to those without anterior sextants. According to Yu et al. [37], the anterior teeth play a vital role in dental esthetics and personal image due to their physiological and psychological importance. Thus, the implantation of anterior teeth can significantly improve patient OHRQoL [37].

The balanced distribution of teeth in the arches (≥10 teeth in each arch) positively affected SOH and IDP, which is similar to data reported by Damyanov et al. [1]. Likewise, having ≥10 teeth in each arch was the most important dental aspect with regard to discriminating impact on OHRQoL among Chinese adults [3]. These findings lend support to previous conclusions regarding the importance of the distribution of the teeth to oral functions [15] as well as OHRQoL [2].

It is also clear that the inclusion of level VI to the functional classification system of dentitions proposed by Nguyen et al. [16] had a significant positive effect on SOH and smiling/speaking. This underscores the importance of periodontal status to OHRQoL, as described elsewhere [22–24], since such an influence is not found on the previous level (level V - molars).

In the multiple regression models, only SOH, an absence of overall impact and smiling/speaking were affected by the definitions of FD after adjustments for the controlling variables (monthly household income, schooling, self-rated need for treatment, toothache/dental pain in previous 6 months, self-rated need for dentures or to change dentures, reason for last dental appointment and gender). Similar results are reported for Chinese adults [3], for whom dental conditions lost the association with poorer OHRQoL in the presence of controlling (demographic and socioeconomic) variables. The inclusion of these variables in the models is justified by the influence of demographic and socioeconomic characteristics on OHRQoL as well as the association between such variables and dental conditions [2, 4, 11, 32]. In contrast, Damyanov et al. [1] found a significant association between dental condition and general satisfaction with oral health, esthetics and chewing function even after the incorporation of controlling variables (demographic-socioeconomic characteristics, use of services and behavior). The possible explanation for these differences is the better living conditions found in Bulgaria in comparison to Brazil and China, at least with regard to human development and the distribution of wealth. In the ranking of the Human Development Index (HDI), Bulgaria is in a more favorable position (HDI = 0.77) than Brazil (HDI = 0.72) and China (HDI = 0.69). The distribution of income, which is measured using the Gini coefficient, is also more equitable in Bulgaria than Brazil [38]. Thus, contextual social inequalities and their effects on OHRQoL do not seem to be a reality in Bulgaria like it was seen in Brazil [11].

More complete definitions (WDT, FD_Class5_ and FD_Class6_) than merely the number of teeth present (FDWHO) exerted a significant positive effect on SOH and the absence of overall impact. This finding can contribute to the establishment of a new definition of FD that incorporates both normative and subjective aspects. Although FDWHO only remained associated with smiling/speaking in the present investigation, the authors of a study conducted involving adults in Finland found that the presence of this condition resulted in a lower chance of the occurrence of impacts in the categories of “reasonably” and “very often” [32]. However, the evidence regarding the positive association between dentitions that meet a greater number of functionality criteria and OHRQoL seems to be more consistent. Such dentitions include the SDA evaluated in comparison to prosthetic replacement [12] and the SDA with a minimum number of POPs [33]. These findings are in line with the conclusion that tooth loss is negatively associated with OHRQoL, which is further compromised in the absence of POPs and anterior teeth [2]. However, the authors state that the impact of the location and distribution of teeth remains an object for future investigations, which lends further strength to the present findings.

The implications for public health are well known. Oral healthcare interventions are burdensome and the demand for such care tends to increase with the increase in the proportion of elderly individuals in the population [2], which is a trend seen throughout the world. The demand for treatment is not well correlated with treatment needs determined based on normative criteria and it has been recognized that objective measures of adverse health conditions are not good predictors of demand [2]. Thus, as the resources for dental treatment have become increasingly scarce, new paradigms for evaluating oral health have been developed [2] and need to be employed in public services. The rationale for this is the prioritization of scarce financial resources for patients that can benefit from more specific therapies [2]. Administrators in the public health setting should direct resources toward patients who are dissatisfied with their oral health status [2]. This philosophy is particularly relevant when the objective of treatment is not curative and the goal is to reduce morbidity associated with chronic conditions [2], such as dental caries and periodontal disease. Such reflections are important to the context of public health systems, especially in Brazil, where the failure to meet the large demand, especially among adults, is reflected in dental services centered on technique and normative evaluations rather than the perceptions and values of patients. Results that reached to this same point of view strongly suggest that a non-negligible contingent of adults may do without dental prosthesis, despite having several missing teeth [13].

Despite the contributions of the present findings, future evaluations should be conducted in the form of qualitative studies to identify how the presence of different definitions of FD is perceived by the population [39]. A multidimensional assessment that incorporates a four-dimensional OHRQoL model consisting of oral function, oro-facial pain, oro-facial appearance and psychosocial impact [40] and includes the effect of prosthetic replacement is being outlined by the authors for use in future studies.

## Conclusions

Satisfaction with oral health and impacts on daily performance among Brazilian adults were significantly associated with different definitions of functional dentition. The influence of WDT, FD_Class5_ and FD_Class6_ on more aspects related to OHRQoL in comparison to FDWHO demonstrates the need to establish a broader definition of *functional dentition*. The incorporation of subjective aspects into decision-making processes in public services regarding both the planning of individual treatments and the formulation of public policies could contribute to the better application of resources. This measure will allow approaching the principal of equity and improving the quality of life of patients who utilize public services.

## Abbreviations

CAL: Clinical attachment loss
CIs: Confidence intervals
CPI: Community periodontal index
DMFT: Decayed, missing and filled teeth
FD: Functional dentition
FD_Class5_: Functional dentition classified by five levels
FD_Class6_: Functional dentition classified by six levels
FDWHO: Functional dentition established by the World Health Organization
IDP: Impacts on daily performance
NOHS: National oral health survey
OHRQoL: Oral health-related quality of life
OIDP: Oral impacts on daily performance
POPs: Posterior occluding pairs
SDA: Shortened dental arch
SOH: Satisfaction with oral health
TMD: Temporomandibular disorders
WDT: Well-distributed teeth
WHO: World Health Organization

## References

[1] Damyanov ND, Witter DJ, Bronkhorst EM, Creugers NH (2013). Satisfaction with the dentition related to dental functional status and tooth replacement in an adult Bulgarian population: a cross-sectional study. Clin Oral Investig.

[2] Gerritsen AE, Allen PF, Witter DJ, Bronkhorst EM, Creugers NH (2010). Tooth loss and oral health-related quality of life: a systematic review and meta-analysis. Health Qual Life Outcomes.

[3] Zhang Q, Witter DJ, Gerritsen AE, Bronkhorst EM, Creugers NH (2013). Functional dental status and oral health-related quality of life in an over 40 years old Chinese population. Clin Oral Investig.

[4] Åstrøm AN, Haugejorden O, Skaret E, Trovik TA, Klock KS (2006). Oral impacts on daily performance in Norwegian adults: the influence of age, number of missing teeth, and socio-demographic factors. Eur J Oral Sci.

[5] Hwang SJ, Patton LL, Kim JH, Kim HY (2012). Relationship between oral impacts on daily performance and chewing ability among independent elders residing in Daejeon City, Korea. Gerodontology.

[6] Abegg C, Fontanive VN, Tsakos G, Davoglio RS, Oliveira MMC (2015). Adapting and testing the oral impacts on daily performances among adults and elderly in Brazil. Gerodontology.

[7] Locker D (1988). Measuring oral health: a conceptual framework. Community Dent Health.

[8] Adulyanon S, Vourapukjaru J, Sheiham A (1996). Oral impacts affecting daily performance in a low dental disease Thai population. Community Dent Oral Epidemiol.

[9] Masalu JA, Astrøm AN (2003). Applicability of an abbreviated version of the oral impacts on daily performances (OIDP) scale for use among Tanzanian students. Community Dent Oral Epidemiol.

[10] Srisilapanan P, Sheiham A (2001). The prevalence of dental impacts on daily performances in older people in northern Thailand. Gerodontology.

[11] Vettore MV, Aqeeli A (2016). The roles of contextual and individual social determinants of oral health-related quality of life in Brazilian adults. Qual Life Res.

[12] Wolfart S, Muller F, Gerss J, Heyedcke G, Marre B, Boning K, Wostmann B, Kern M, Mundt T, Hannak W (2014). The randomized shortened dental arch study: oral health-related quality of life. Clin Oral Investig.

[13] Antunes JL, Tan H, Peres KG, Peres MA (2016). Impact of shortened dental arches on oral health-related quality of life. J Oral Rehabil.

[14] WHO Expert Coomittee on Recent Advances in Oral Health. Recent advances in oral health: report of a WHO expert committee. In: WHO Technical Report Series. Geneva: World Health Organization; 1992: 38. 1462607

[15] Gotfredsen K, Walls AW (2007). What dentition assures oral function?. Clin Oral Implants Res.

[16] Nguyen TC, Witter DJ, Bronkhorst EM, Pham LH, Creugers NHJ (2011). Dental functional status in a southern Vietnamese adult population—a combined quantitative and qualitative classification system analysis. Int J Prosthodont.

[17] Chalub LLFH, Ferreira RC, Vargas AM (2016). Functional, esthetical, and periodontal determination of the dentition in 35- to 44-year-old Brazilian adults. Clin Oral Investig.

[18] Chalub LLFH, Martins CC, Ferreira RC, Vargas AM (2016). Functional dentition in Brazilian adults: an investigation of social determinants of health (SDH) using a multilevel approach. PLoS One.

[19] Nguyen TC, Witter DJ, Bronkhorst EM, Gerritsen AE, Creugers NHJ (2011). Chewing ability and dental functional status. Int J Prosthodont.

[20] Zhang Q, Witter DJ, Bronkhorst EM, Creugers NH (2013). Chewing ability in an urban and rural population over 40 years in Shandong Province, China. Clin Oral Investig.

[21] Okada T, Ikebe K, Inomata C, Takeshita H, Uota M, Mihara Y, Matsuda K, Kitamura M, Murakami S, Gondo Y (2014). Association of periodontal status with occlusal force and food acceptability in 70-year-old adults: from SONIC study. J Oral Rehabil.

[22] Cunha-Cruz J, Hujoel PP, Kressin NR (2007). Oral health-related quality of life of periodontal patients. J Periodontal Res.

[23] Needleman I, McGrath C, Floyd P, Biddle A (2004). Impact of oral health on the life quality of periodontal patients. J Clin Periodontol.

[24] Costa FO, Miranda Cota LO, Pereira Lages EJ, Vilela Camara GC, Cortelli SC, Cortelli JR, Costa JE, Medeiros Lorentz TC (2011). Oral impact on daily performance, personality traits, and compliance in periodontal maintenance therapy. J Periodontol.

[25] Ministery of Health (BR). SB Brasil 2010: Pesquisa Nacional de Saúde Bucal: resultados principais. In. Edited by Secretaria de Vigilância em Saúde. Secretaria de Atenção à Saúde. Departamento de Atenção Básica. Coordenação Nacional de Saúde Bucal.. Brasília: Ministery of Health (BR); 2011:92.

[26] Silva NN, Roncalli AG (2013). Sampling plan, weighting process and design effects of the Brazilian oral health survey. Rev Saude Publica.

[27] Roncalli AG, Silva NN, Nascimento AC, Freitas CHSM, Casotti E, Peres KG, Ld M, Peres MA, Freire MCM, Cortes MIS (2012). Relevant methodological issues from the SBBrasil 2010 project for national health surveys. Cad Saúde Pública.

[28] Ministery of Health (BR). Projeto SB Brasil 2003: condições de saúde bucal da população brasileira 2002-2003: resultados principais. In. Edited by Secretaria de Atenção à Saúde. Departamento de Atenção Básica.. Brasília: Ministery of Health (BR); 2004: 51.

[29] World Health Organization. Oral Health Surveys: Basic Methods, 4th ed edn. Geneva: World Health Organization; 1997.

[30] Borrell LN, Papapanou PN (2005). Analytical epidemiology of periodontitis. J Clin Periodontol.

[31] Ministery of Health (BR) SB Brasil 2010 - Pesquisa Nacional de Saúde Bucal: Manual da Equipe de Campo. In. Edited by Secretaria de Vigilância à Saúde, Secretaria de Atenção à Saúde, Departamento de Atenção Básica, Coordenação Nacional de Saúde Bucal Brasília: Ministério da Saúde; 2009.

[32] Lahti S, Suominen-Taipale L, Hausen H (2008). Oral health impacts among adults in Finland: competing effects of age, number of teeth, and removable dentures. Eur J Oral Sci.

[33] Tan H, Peres KG, Peres MA (2015). Do people with shortened dental arches have worse oral health-related quality of life than those with more natural teeth? A population-based study. Community Dent Oral Epidemiol.

[34] Reissmann DR, Heydecke G, Schierz O, Marre B, Wolfart S, Strub JR, Stark H, Pospiech P, Mundt T, Hannak W (2014). The randomized shortened dental arch study: temporomandibular disorder pain. Clin Oral Investig.

[35] Brennan DS, Spencer AJ, Roberts-Thomson KF (2008). Tooth loss, chewing ability and quality of life. Qual Life Res.

[36] Ervin RB, Dye BA (2012). Number of natural and prosthetic teeth impact nutrient intakes of older adults in the United States. Gerodontology.

[37] Yu SJ, Chen P, Zhu GX (2013). Relationship between implantation of missing anterior teeth and oral health-related quality of life. Qual Life Res.

[38] United Nations Development Programme. Human Development Report 2011 - Sustainability and Equity: A Better Future for All. In. Basingstoke: United Nations Development Programme; 2011: 176.

[39] Locker D, Allen F (2007). What do measures of ‘oral health-related quality of life’ measure?. Community Dent Oral Epidemiol.

[40] John MT, Rener-Sitar K, Baba K, Celebic A, Larsson P, Szabo G, Norton WE, Reissmann DR (2016). Patterns of impaired oral health-related quality of life dimensions. J Oral Rehabil.

